# Supplementary material for: Assessing the multifunctionality of service crops in mediterranean vineyards using a functional trait approach
Source: PLoS One. 2026 Feb 23;21(2):e0343005. doi: 10.1371/journal.pone.0343005 (PMC12928470; doi:10.1371/journal.pone.0343005)
Supplement: S1 Table — (PDF) [file pone.0343005.s001.pdf]

S1 Table. Sown species chosen for the experimentation and their respective botanical family.

Name	Family
Achillea millefolium	Asteraceae
Brassica carinata	Brassicaceae
Dactylis glomerata	Poaceae
Festuca ovina	Poaceae
Medicago lupulina	Fabaceae
Medicago sativa	Poaceae
Phacelia tanacetifolia	Hydrophylaceae
Plantago coronopus	Plantaginae
Poterium sanguisorba	Rosaceae
Secale cereale	Poaceae
Trifolium fragiferum	Fabaceae
xTriticosecale	Poaceae
Vicia villosa	Fabaceae
