# Supplementary material for: Assessing the multifunctionality of service crops in mediterranean vineyards using a functional trait approach
Source: PLoS One. 2026 Feb 23;21(2):e0343005. doi: 10.1371/journal.pone.0343005 (PMC12928470; doi:10.1371/journal.pone.0343005)
Supplement: S2 Table — (PDF) [file pone.0343005.s002.pdf]

S2 Table. Composition of each plant community, relative biomass of each species (%) and total biomass of the community (t ha^{-1}).

Code	Species	Relative biomass (%)	Total biomass (t ha^{-1})
Am4	Achillea millefolium	38.6	2.0
	Galium parisiense	24	
	Erodium cicutarium	20	
	Carduus pycnocephalus	11.3	
	Filago pyramidata	6.1	
Dg4	Dactylis glomerata	37.3	1.4
	Carduus pycnocephalus	32.1	
	Erodium cicutarium	11.3	
	Medicago sativa	9.5	
	Erodium malacoides	5.3	
	Sonchus asper	4.4	
Ml2	Medicago lupulina	80.1	3.0
	Carduus pycnocephalus	19.9	
Ms1	Medicago sativa	58.5	1.9
	Daucus carota	34.3	
	Erodium malacoides	7.2	
Pc2	Plantago coronopus	85.3	2.6
	Filago pyramidata	8.9	
	Geranium rotundifolium	4.2	
	Galium parisiense	1.6	
Pt4	Phacelia tanacetifolia	74.2	2.7
	Medicago sativa	17.6	
	Geranium rotundifolium	8.2	
Sm1b	Sanguisorba minor	34.3	1.8
	Cirsium arvense	18.6	
	Picris echioides	18	
	Erodium malacoides	8.6	
	Galium parisiense	7.6	
	Daucus carota	6	
	Erodium cicutarium	5.5	
	Sonchus asper	1.5	
Sm3a	Sanguisorba minor	27.1	1.1
	Galium parisiense	34.8	
	Erodium malacoides	20	
	Sonchus asper	15.7	
	Daucus carota	2.5	
Sp2	Geranium rotundifolium	37.6	2.3
	Galium parisiense	31.3	
	Carduus pycnocephalus	10.7	
	Cirsium vulgare	10.6	
	Sonchus asper	9.7	

Ts2	Triticosecale	45.7	2.4
	Medicago sativa	25.3	
	Sonchus asper	14.9	
	Daucus carota	8.3	
	Carduus pycnocephalus	5.7	
Ts5	Triticosecale	90.3	1.4
	Cirsium arvense	9.7	
Am4	Achillea millefolium	40.5	2.0
	Galium parisiense	19.5	
	Scorpiurus subvillosus	15.3	
	Erodium cicutarium	10.6	
	Filago pyramidata	7.2	
	Carduus pycnocephalus	4.1	
	Picris echioides	2.8	
Dg1	Dactylis glomerata	61	2.8
	Carduus pycnocephalus	39	
Ml3	Medicago lupulina	78.3	1.8
	Galium parisiense	21.7	
Ms2	Medicago sativa	34	2.1
	Erodium cicutarium	34.9	
	Crepis sancta	16.2	
	Galium parisiense	14.1	
	Picris echioides	0.8	
Pc2	Plantago coronopus	100	3.4
Pt3	Phacelia tanacetifolia	100	2.6
Sp4	Erodium ciconium	42.1	2.8
	Rostraria cristata	12.3	
	Crepis sancta	11.8	
	Erodium cicutarium	8.2	
	Erigeron canadensis	6.7	
	Picris echioides	5.4	
	Galium parisiense	5.3	
	Filago pyramidata	3.7	
	Veronica persica	2.5	
	Sonchus asper	2	
Vv2	Vicia villosa	100	1.9
Am3	Achillea millefolium	56.5	2.2
	Carduus pycnocephalus	20.1	
	Erodium cicutarium	12.2	
	Galium parisiense	11.2	
Bc4	Brassica carinata	82.5	2.1
	Cerastium glomeratum	8.4	
	Erodium cicutarium	5.9	
	Galium parisiense	3.2	

Dg4	Dactylis glomerata	30	2.4
	Medicago orbicularis	21.4	
	Carduus pycnocephalus	13.4	
	Malva sylvestris	11	
	Erodium cicutarium	7.4	
	Cerastium glomeratum	3.6	
	Galium parisiense	3.5	
	Daucus carota	2.8	
	Picris echioides	1.7	
	Euphorbia segetalis	1.6	
	Trifolium campestre	1.4	
	Veronica persica	0.9	
	Sonchus asper	0.7	
	Convolvulus arvensis	0.6	
Fo2b	Festuca ovina	29.1	1.8
	Erodium cicutarium	27	
	Carduus pycnocephalus	16.4	
	Picris echioides	10.2	
	Plantago coronopus	6.4	
	Daucus carota	5.5	
	Veronica persica	2.7	
	Galium parisiense	2.6	
Fo4a	Festuca ovina	16.4	1.1
	Diplotaxis erucoides	19.2	
	Carduus pycnocephalus	15.7	
	Sonchus asper	8.1	
	Plantago coronopus	7.4	
	Convolvulus arvensis	7.3	
	Galium parisiense	7.1	
	Daucus carota	5.1	
	Picris echioides	4.1	
	Anagallis arvensis	3.5	
	Erodium cicutarium	3	
	Geranium rotundifolium	1.7	
	Crepis sancta	1.4	
Fo4c	Festuca ovina	31.7	0.9
	Plantago coronopus	29.9	
	Picris echioides	12.4	
	Galium parisiense	7.6	
	Scorpiurus subvillosus	6.5	
	Diplotaxis erucoides	4.9	
	Anagallis arvensis	4.7	
	Cirsium arvense	2.1	

Ml4	Medicago lupulina	91.4	2.4
	Erodium cicutarium	5.8	
	Picris echioides	2.8	
Ms1	Medicago sativa	37	2.8
	Erodium malacoides	16.2	
	Filago pyramidata	11.2	
	Cerastium glomeratum	10.5	
	Veronica persica	9.6	
	Galium parisiense	7.8	
	Carduus pycnocephalus	3.3	
	Geranium rotundifolium	2.4	
	Scorpiurus subvillosus	2	
Pc3	Plantago coronopus	93.4	4.1
	Galium parisiense	6.6	
Pt1	Phacelia tanacetifolia	100	3.3
Sc3b	Secale cereale	100	1.8
Sc4b	Secale cereale	97.6	2.2
	Festuca ovina	2.4	
Sm1	Sanguisorba minor	42.7	2.1
	Scorpiurus subvillosus	46.2	
	Galium parisiense	11.1	
Sp3	Erigeron canadensis	35.5	2.0
	Cerastium glomeratum	34.1	
	Medicago sativa	17.2	
	Filago pyramidata	5.2	
	Trifolium campestre	5	
	Galium parisiense	3	
Tf1a	Medicago orbicularis	59.6	4.4
	Erodium malacoides	18.3	
	Veronica persica	11.3	
	Galium parisiense	6.6	
	Picris echioides	3.9	
	Galactites elegans	0.3	
Tf2b	Trifolium fragiferum	67.4	4.8
	Trifolium campestre	8.9	
	Diplotaxis erucoides	6.2	
	Picris echioides	5.1	
	Sonchus asper	4.4	
	Erodium malacoides	3.7	
	Daucus carota	2.7	
	Galium parisiense	1.6	

Tf3c	Trifolium fragiferum	54.7	5.8
	Trifolium campestre	12.3	
	Erodium cicutarium	8.1	
	Veronica persica	7.7	
	Galium parisiense	7.7	
	Medicago orbicularis	3.3	
	Sonchus asper	3.3	
	Cerastium glomeratum	1.5	
	Picris echioides	0.8	
	Malva sylvestris	0.5	
Vv3a	Vicia villosa	100	14.7
Vv4b	Vicia villosa	100	12.7
