# Supplementary material for: Assessing the multifunctionality of service crops in mediterranean vineyards using a functional trait approach
Source: PLoS One. 2026 Feb 23;21(2):e0343005. doi: 10.1371/journal.pone.0343005 (PMC12928470; doi:10.1371/journal.pone.0343005)
Supplement: S3 Fig — (PDF) [file pone.0343005.s003.pdf]

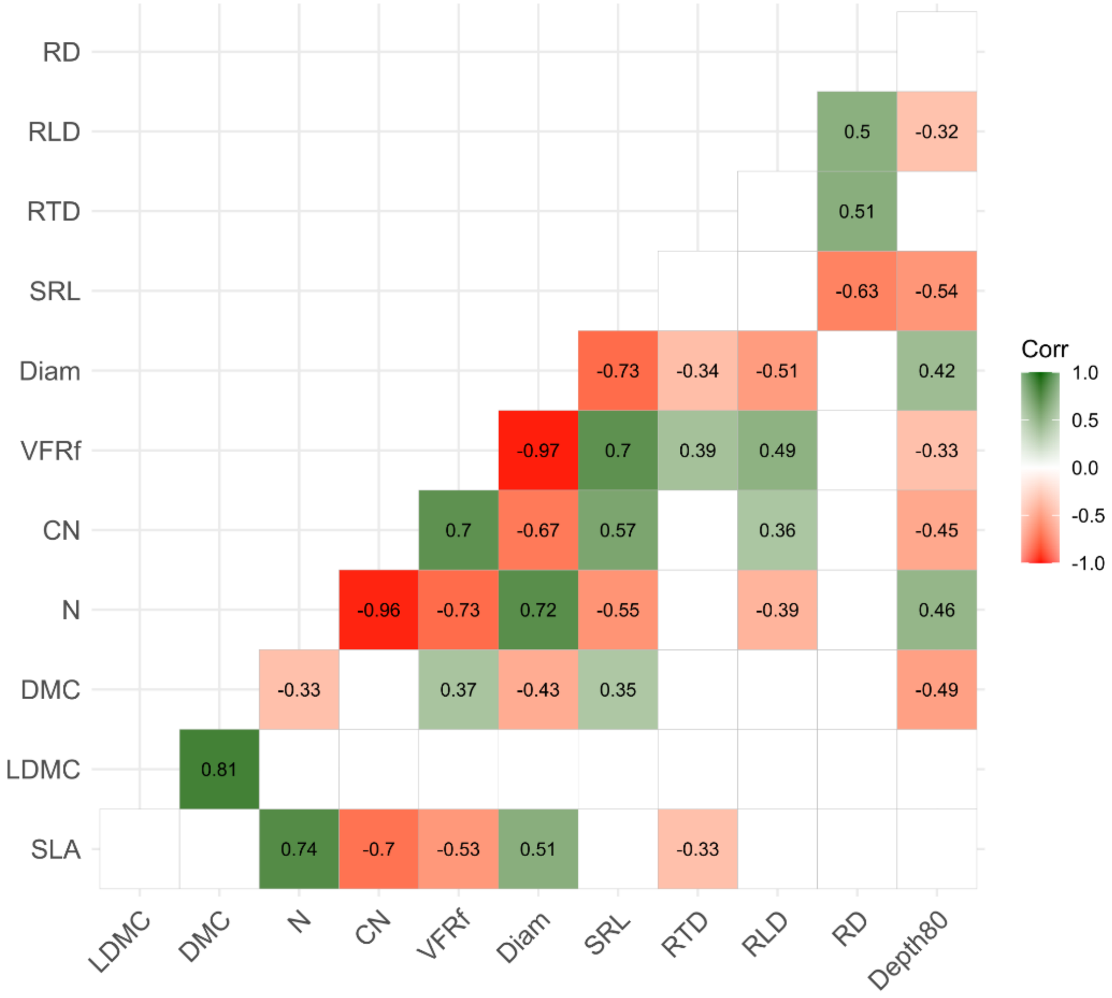


**S3 Figure. Pearson correlation coefficient matrix of functional markers of the communities (n=38).** 'Corr' indicates the value of the correlation coefficient along with a color gradient that indicates its strength. Only significant values (p<0.05) are presented. SLA: Specific Leaf Area. DMC: Dry Matter Content. LDMC: Leaf Dry Matter Content. N: service crop Nitrogen content. CN: service crop C to N ratio. VFRf: Very Fine Root Fraction of the total soil profile. Diam: root mean diameter; SRL: specific root length; RTD: root tissue density; RMD: root mass density; RLD: root length density. RD: root density. Depth80: the depth to reach 80% of total root length.
