# Supplementary material for: Assessing the multifunctionality of service crops in mediterranean vineyards using a functional trait approach
Source: PLoS One. 2026 Feb 23;21(2):e0343005. doi: 10.1371/journal.pone.0343005 (PMC12928470; doi:10.1371/journal.pone.0343005)
Supplement: Attachment — Submitted filename: Response to Reviewers_R1.docx [file pone.0343005.s005.docx]

**Response to Reviewers**

References to line numbers correspond to the revised manuscript with track changes.

| 1. Please ensure that your manuscript meets PLOS ONE's style requirements, including those for file naming. | We have modified the style of the manuscript to meet the journal requirements. |
| --- | --- |
|  |  |
| 2. We noticed you have some minor occurrence of overlapping text with the following previous publication(s), which needs to be addressed: |  |
| <https://linkinghub.elsevier.com/retrieve/pii/S1360138522000863> | We reformulated the reference to paper 47 from line 551-554 |
| <https://hal.science/hal-01614417v2/document> | References to paper 44 were reformulated from line 103 to 110 |
| <https://www.frontiersin.org/journals/sustainable-food-systems/articles/10.3389/fsufs.2020.564197/full> | In lines 65-66, we reformulated the argument presented in this paper |
| In your revision ensure you cite all your sources (including your own works), and quote or rephrase any duplicated text outside the methods section. Further consideration is dependent on these concerns being addressed. | We rephrased l 127-129 |
|  |  |
| 3. Thank you for stating the following financial disclosure:  “LG, AM: FertilCrop project, FP7 ERA-Net program CORE Organic Plus”  Please state what role the funders took in the study.  If the funders had no role, please state: "The funders had no role in study design, data collection and analysis, decision to publish, or preparation of the manuscript."  If this statement is not correct you must amend it as needed.  Please include this amended Role of Funder statement in your cover letter; we will change the online submission form on your behalf. | Actually, the funders had no role in study design, data collection and analysis, decision to publish, or preparation of the manuscript.  This has been precised in the cover letter |
|  |  |
| 4. Thank you for stating the following in the Acknowledgments Section of your manuscript:  “[…]”  We note that you have provided funding information that is currently declared in your Funding Statement. However, funding information should not appear in the Acknowledgments section or other areas of your manuscript. We will only publish funding information present in the Funding Statement section of the online submission form. Please remove any funding-related text from the manuscript and let us know how you would like to update your Funding Statement. Currently, your Funding Statement reads as follows: “LG, AM: FertilCrop project, FP7 ERA-Net program CORE Organic Plus” Please include your amended statements within your cover letter; we will change the online submission form on your behalf. | We have removed funding stratement from the acknowledgement section. |
|  |  |
| 5. When completing the data availability statement of the submission form, you indicated that you will make your data available on acceptance. We strongly recommend all authors decide on a data sharing plan before acceptance, as the process can be lengthy and hold up publication timelines. Please note that, though access restrictions are acceptable now, your entire data will need to be made freely accessible if your manuscript is accepted for publication. This policy applies to all data except where public deposition would breach compliance with the protocol approved by your research ethics board. If you are unable to adhere to our open data policy, please kindly revise your statement to explain your reasoning and we will seek the editor&apos;s input on an exemption. Please be assured that, once you have provided your new statement, the assessment of your exemption will not hold up the peer  review process. | The dataset was made available on the CIRAD Dataverse on our joint research unit ABSYS on the following link: <https://dataverse.cirad.fr/dataset.xhtml?persistentId=doi:10.18167/DVN1/0TKB41> |
|  |  |
| 6. Please include captions for your Supporting Information files at the end of your manuscript, and update any in-text citations to match accordingly. Please see our Supporting Information guidelines for more information: http://journals.plos.org/plosone/s/supporting-information. | We have add supporting informations titles at the end of the manuscript and followed your guideline for their names, citations and formats. |
|  |  |
| **Reviewer #1** | |
| 1. I believe the manuscript would benefit from a deeper discussion in the introduction and discussion sections regarding the “afterlife effects” of leaf functional traits and their influence on multiple litter functions. This would be particularly relevant for interpreting your results related to nitrogen fluxes and soil conservation. In this regard, I strongly recommend considering the following reference: Dias, A.T.C., Cornelissen, J.H.C. & Berg, M.P. (2017). Litter for life: assessing the multifunctional legacy of plant traits. Journal of Ecology, 105(5), 1163–1168. https://doi.org/10.1111/1365-2745.12763 | L. 80 we already refer to leaf functional traits in relation to litter decomposition: The leaf nitrogen content is also a marker of the acquisitive strategy of species and relates to the litter decomposition and mineralization [19,20,24] |
| 2. I would recommend re-evaluating the use of the term “runoff reduction” as one of the assessed functions. Since actual runoff was not measured directly in your study, and rather inferred from total cover rate of plant community, it would be more accurate to describe this as a proxy. As runoff can also be strongly influenced by factors such as slope and soil texture, I suggest acknowledging these limitations more explicitly in the text. | We kept the term "Runoff Reduction" for our indicator, but added some precisions about the use of the cover rate as a proxy as suggested. We added these precisions in the material and methods section 2.2.3 (sub section Runoff Reduction) l 244-245, and in the first section of discussion (l.512-513) |
| 3. Although the manuscript includes nearly a hundred references, a few of them could be better aligned with specific statements. I suggest incorporating the following references, which may provide more precise support: o Lines 55–56: Castillo-Figueroa, D., Soler-Marín, D., & Posada, J.M. (2025). Functional Traits and Species Identity Drive Decomposition Along a Successional Gradient in Upper Andean Tropical Forests. Biotropica, 57: e13425. https://doi.org/10.1111/btp.13425 o Line 413: Álvarez-Rogel, J., Peñalver-Alcalá, A., & González-Alcaraz, M.N. (2022). Spontaneous vegetation colonizing abandoned metal(loid) mine tailings consistently modulates climatic, chemical and biological soil conditions throughout seasons. Science of the Total vironment, 838, 155945. https://doi.org/10.1016/j.scitotenv.2022.155945 o Lines 489–490:  Castillo-Figueroa, D., González-Melo, A., & Posada, J.M. (2023). Wood density is related to aboveground biomass and productivity along a successional gradient in upper Andean tropical forests. Frontiers in Plant Science, 14, 1276424. https://doi.org/10.3389/fpls.2023.1276424 o Line 492: Lavorel, S., & Garnier, E. (2002). Predicting changes in community composition and ecosystem functioning from plant traits: revisiting the Holy Grail. Functional Ecology, 16, 545–556. https://doi.org/10.1046/j.1365-2435.2002.00664.x o Lines 532–533: Kümmerer, R., Heuermann, D., Laidig, F., Piepho, H.-P., & Bauer, B. (2025). Strategies to develop simple multi-species cover crop mixtures to enhance aboveground biomass and quality. Field Crops Research, 333, 110014. https://doi.org/10.1016/j.fcr.2025.110014 | We added the two last references suggested in the manuscript, and Alvarel et al. 2022 in complement to Alvarel et al. 2021 as suggested. Lavorel & Garnier (2002) was added l.605 as suggested, and also in the introduction of the manuscript l.73. Kummerer et al. (2025) was added l.645 as suggested. We chose to not include the first three references due to i) their specific context (Andean tropical forest, woody species), and ii) the fact that the paper already contains a high number of scientific references. |
| 4. Finally, I think the manuscript would gain additional relevance if the main findings were briefly placed within a broader land-use management context. Although I understand that policy application may not be the central focus of the study, one or two sentences connecting your findings to decision-making processes or practical implications for sustainable agriculture would add value and accessibility for broader audiences. | We added a paraghaph as suggested by the reviewer at the end of the discussion, l.670-678 |
|  |  |
| **Reviewer #2:** | |
| Title: Adjust the term ‘crops’, since throughout the document emphasis has been placed on agrosystems, ecosystems, vineyard landscapes, and plant communities. Could be: agrosystems with grapevines. | We are not sure to understand the suggestion. The term "service crops" is used in the title as the paper focus on managed plant communities in vineyards inter-rows (either spontaneous or sown), grown to provide ecosystem services (see Garcia et al. 2018). We think the title is adapted to the scope of the paper, and would like to keep it unchanged. |
| **ABSTRACT** |  |
| Line 12-20: It is suggested to focus on the problem and justification. | We reformulated the abstract in order to focus on the problem and justification of the approach l 17-l19 |
| Summarize. Give more relevance to your findings. |  |
| It is suggested to include the treatments and experimental design. Likewise, mention which variables were measured. Statistical analysis too. | We added information about the treatments and experimental design as suggested, mentioned the ecosystem functions we focused on, and added some information about the statistics used l.24-32. |
| Line 27: Correctly denote the regression coefficient. | Corrected |
| Line 32: How was sustainability measured in the agrosystem? | The last sentence of the abstract is a perspective. In the paper we measured the multifunctionality of diverse plant communities, the sustainability of the agrosystem was not measured per se. |
| **INTRODUCTION** |  |
| Line 37-67: It is suggested that some numerical data be added to strengthen the variables in question. For example, how much productivity increases under these diversified systems. | We added a quantification of productivity increase l. 61-62 "; for example, Marini et al. (2020) estimated annual yield increases of approximately 860 kg ha⁻¹ for winter cereals and 390 kg ha⁻¹ for spring cereals thanks to crop diversification, respectively. " |
| Line 94: What does ES mean? | ES stands for "ecosystem services". The abbreviation was replaced (l.123-124) |
| Line 94-95: Clarify what is a synthetic indicator? | We clarified this concept (l.124) |
| Line 97: In what phenological states of the grapevine plant were these parameters measured? | These parameters were measured at grapevine budburst (see l. 165-167) |
| It is suggested to include the economic importance of this type of vineyard system and the proportion in which it exists in the country. Also, in terms of public policies, what has been done for the management of these systems? It is suggested to include the knowledge gap regarding the variables and the research subject. | As this paper does not focus on an economical study but on indicators related to biophysical ecosystem services, we think it’s not necessary to add this information in the introduction |
| **Material and methods** |  |
| It is suggested to add the name of the equipment and instruments that were used to measure the variables in question (brand, model, condition and country of manufacture).  It is suggested to add the type of climate and soil at the experimental site.  It is uggested to add a representative Figure of how the treatments and replicas were distributed.   Explain why these 13 plant species were used for the experiment. Maybe the answer is on line 120, but there's no connection between the paragraphs. Clarify this point.  Based on what method were the 13 botanical families used in the experiment determined? Also specify whether the experiment was established in the open field or under greenhouse conditions. | We indicated the name of equipment and brands when it was possible, for example regarding to the scanner or software used for root trait measurements. We added the information regarding the precision balance used. For the other equipment, it was either home-made (e.g. the quadrats), very basic (e.g. cutters to sample plant biomass), or unknown (nitrogen analisis made in an external laboratory).  The climate and soil are described in Garcia et al. (2019, 2020), and we indicated in this manuscript the cumulated rainfall l 144  We merged the two first paragraphs of the Material and Methods section to paste the explanation of the choice of the 13 species to the first mention of this. Species were chosen to have a diversity of botanical families, life cycles, and growth rate, and were determined based on litterature analysis, this point was added to the M&M l.162  The experiment was established in a vineyard (i.e. open field), it’s indicated in the first sentence of the M&M "The experimental vineyard was located near Montpellier […]" l.143 |
| Line 124: Were the quadrants established permanently or temporarily? | We do not understand the question, the quadrats were placed on the different plant communities at grapevine budburst to sample the aboveground biomass and simultaneously measure the other indicators. |
| Line 129: It is suggested to add the age of the plant. | The age of the grapevine is indicated in Garcia et al. (2019, 2020), we added the information at the beginning of the M&M section l.143 |
| Line 153: Specify the number of subsamples of herbaceous roots. | Done, l.201 |
| Line 172: Please specify why it was not calculated for all 5 soil layers? | We used the five soil layers to calculate the depth at which each plant community reached 80% of its total root length. Other root functional markers were calculated in the 0-20 cm soil layer, corresponding to the mean depth at which plant communities reached 80% of their total root length across all plant communities (Garcia et al. 2020). The justification was added at the end of the paragraph l.222-224 |
| In Table 1, we suggest adding the authors from which these variables were taken. Also, in the first column, describe the acronym in full and include the acronym in parentheses. | Table 1 was modified according to reviewer's comment |
| Line 180: add at what age of the plants these variables were taken. | The vineyard was planted in 2008, the information was added in the site description. |
| Line 198: What is the degree of purity of ethanol? | The information was added (96°) l.256 |
| Line 222: Justify why up to 1 m deep? and not 2 meters. Support with a bibliographic citation. | The study focused on measuring the effects of annual service crops, which root system did not reach such a deep soil layer (see Garcia et al. 2020 or the functional marker Depth80 used in this paper) |
| Line 248-249: For what subsequent statistical analysis were these weighted means used? | The Community Weighted Means were calculated for each trait to reflect the mean functional structure of the plant communities that were composed by several species (see Garnier et al. 2004, Pakeman and Quested 2007). For a trait, the resulting value corresponds to a "mean trait" at the community scale. |
| Line 257-258: Why was a dendrogram not made with Euclidean or Gower ditances | Hierarchical clustering on principal components (HCPC), performed using the FartoMineR package, actually involves constructing a dendrogram using the Euclidean distance method. Clustering is then performed based on this dendrogram. Lê, S., Josse, J., & Husson, F. (2008). FactoMineR: An R Package for Multivariate Analysis. Journal of Statistical Software, 25(1), 1–18. https://doi.org/10.18637/jss.v025.i01 |
| **Results** |  |
| Line 280: It is suggested to add how many axes add up to 100% of the total variance. | In PCA, too many axes are usually required to achieve 100% of the total variance. One of the objectives of this analysis is to reduce the dimensions of the dataset; therefore, it is important to identify the axes that account for the most variance, rather than all of it. To do so, we observed the decrease of percentage of variance of each component, as suggested by the factomineR package. |
| Line 282-283: The sum of the contributions of each variable is 62.6%. Verify. | The contribution of a variable to the axes is not the same as the percentage of variance explained by the axis. There is no mistake. |
| Line 290-291: What does moderate positive correlation mean? It's better to give values. | Values of correlations were added l.357-360 |
| Line 292: Figure 2A is not in the document. Please verify. | Right. We have now included this figure and modified figure numbers accordingly |
| In Figure 1 separate the words from the supplementary variables. | Modified |
| In Figure 1 add Quadrants I, II, III and IV. | Each point a plant community sampled with 4 quadrats |
| Line 295-297: Specify how the botanical families were distinguished in both axes 1 and 2, since this was to be expected since they were different species | We modified the text to specify that the botanical families were not used to build the clusters but to characterize them (l. 363-364) |
| Line 299: Add the subject of study. | We do not understand the suggestion |
| Line 318: Replace ‘experiment’ with ‘13 plant communities’. Please verify. | Modified |
| Line 371: Add the subject of study. | We do not understand the suggestion |
| Line 373-374: what does resp. mean? | Modified |
| In Figure 4 also separate the words from the variables | Modified |
| In Figure 3B, what do the orange triangles, green circles, and blue squares represent? Label what they correspond to. | These polygones are clusters. This is specified in the legend of the figure. |
| In Figure 6, in the plants, represent the grapevine crop. | As this paper focuses on soil and services crops variables, we prefer not to represent the grapevine. |
| **Discussion** |  |
| In this section, repeat the same subheadings from the Results to improve the study’s flow. It’s not enough to simply state that the results are the same or different from those of other researchers; you must explain why. Please also add the limitations and future work arising from the results of the present study. Why was an economic analysis not included to determine which treatment would be most appropriate to recommend to grapevine growers? | We think it is not adapted to repeat the same subheadings for the discussion section as this section is meant to put the results in perspective with litterature. Regarding the limits, we integrated them in the 4.2 subsection regarding for example the limitations of traits and multufunctionality indexes. Regarding the perspectives of this paper, we developed them in the 4.3 subsection untitled "Design and management of service crop strategies to increase agrosystem multifunctionality". No economical indicator was measured as it was the objective of the study. |
| **Conclusion** |  |
| They must be concise and based on the hypotheses raised. | The conclusion was revised to explicitly refer to the three hypotheses formulated in this study. |
