# Supplementary material for: Assessing the multifunctionality of service crops in mediterranean vineyards using a functional trait approach
Source: PLoS One. 2026 Feb 23;21(2):e0343005. doi: 10.1371/journal.pone.0343005 (PMC12928470; doi:10.1371/journal.pone.0343005)
Supplement: Attachment — Submitted filename: Response to Reviewers_R2.docx [file pone.0343005.s006.docx]

**Response to Reviewers**

References to line numbers correspond to the revised manuscript with track changes.

| **Reviewer #3:** | |
| --- | --- |
| Data are available and can be downloaded from the link provided. Please ensure filenames match filenames given in ReadMe.tab (csv not tab) and files have proper separators (eg you list a .tab file but separators are semicolon). | Yes, we checked the dataverse and now all the filenames match with the names given in the ReadMe.csv, and all files are csv with proper separators, |
| Abstract L41 I don't understand the wording 'determinant to provide soil-based ecosystem functions' | We reformulate the sentence (l.35-36) |
| L51 could you give these as % increases, either as well as or instead of the mass per hectare: it is unclear whether these yield increases are of significance. | We added the information in % increase at the end of the sentence (l.48) |
| L95 I'm not sure six years ago counts as 'recent' any more. Maybe state '2019' instead of 'recent'. | Done (l.89) |
| L132/3 I'm not sure this is an 'on the one hand' situation - you could just say 'both correlations between functions and the level of EFM'. | Done (l.120-121) |
| Reviewer 1 Comment 1: You have not included a reference to this paper; have you considered it? I can't find any mention on line 80 (original or revised) to leaf functionaltraits in relation to litter decomposition - possibly L65 (original) or L72 (revised). It feels like this comment has not been properly addressed. | l65 there are three references linking leaf traits to litter decomposition « The leaf nitrogen content is also a marker of the acquisitive strategy of species and relates to the litter decomposition and mineralization ». However we replaced Handa et al. 2014 with a more recent research (Dias et al. 2017) (l.64-65) |
| Reviewer 1 Comment 2: It still looks like you measured runoff reduction, whereas that's not what you did: you measured cover rate and estimated runoff reduction. I would prefer that throughout you refer to 'cover rate as proxy of runoff reduction' to make it very clear what was measured. | We modified the introductive paragraph of the « Ecosystem functions monitoring and indicators » section to clarify the difference between the functions we targeted, and the indicators that were chosen as proxys of these functions (l.206-210) |
| L145 'literature', spelling mistake | Done (l.132) |
| L148 superscript 2 in m2 | Done |
| L149/150 this does not make sense - please consider the wording. | Done |
| L155 weighed, not weighted | We replace all the « weighted » occurrences by « weighed » in the text |
| L153 It sounds like you did this all on one day, which I presume you did not - that would be a lot of work! Can you please say how long it took to sample (days, weeks?) and how you controlled for the fact that a later sample would mean the intercrops had longer to grow (and therefore would have more biomass)? | Species identification and sampling (plant biomass and soil) were carried within one week. Thus, there were no significant differences in growth between the samples. We added the information in the text (l.142-143) |
| L158 How did you know that species sampled represented 80% of biomass in each quadrat, without harvesting it all and checking? I presume this is species that were *estimated* to be above 80%. | Total aboveground biomass was sampled from all quadrats, and species were separated and weighed separately after drying, to estimate their relative biomass. That allowed us to select the species for trait measurements, so it was not an estimation. This information is written at the beginning of the « Aboveground vegetation sampling and trait measurements » section. |
| L173-5 It might be worth adding a brief explanatory sentence as to why you did this cutting and mixing - was it to control for something, or for some other reason? It feels oddly labour-intensive. | We modified this part to better explain the methodology as suggested (l.159-165) |
| L185-6 I don't understand this sentence. What does 'with the ratio' mean? | We used the ratio of scanned root length to scanned dry biomass to calculate the root length of non-scanned roots. We reformulated the sentence to clarify (l.182-185) |
| L195 I don't understand 'weighted so as non-scanned samples' - do you mean 'weighed as non-scanned samples'? | Yes. We replaced « weighted » by « weighed » througout the whole document. |
| L200-1 Why didn't you use your own measurement of Depth80 here? Feels like that would be better for your own experiment rather than relying on Garcia 2020? | Indeed this study relies on the same experiment than Garcia et al. 2020, but include more indicators and a multifunctionality approach. We did use our own measurement of Depth80, and the mean Depth80 value across all communities to justify the 0-20 cm layer |
| L211 It's unclear what you mean by 'the amount of organic matter restitutions'. | Thanks for the remark, indeed it’s true that this information brings confusion as we did not use « organic matter restitution » as a targeted function. We deleted from the sentence (l.213) |
| L221 ref 51 is simply a quote of ref 60 so I would remove ref 51 from here. Reference 51 in the list of references also has a curious 'cited' date which should be removed. | We removed ref 51 as suggested (L.223) |
| L237 I'm not sure 'pic' is the right word. | Thanks for the observation, we replaced the word with « peak » which is correct (l.240) |
| L254 weighed, not weighted (check all other instances of 'weighted' - I'm not going to list any more) | We replaced all the « weighted » instances with « weighed » as indicated |
| L259 eq2 WS can't have the units mm if BD has g and cm-3 in it? | the true equation is $WS=WC\times D\times(100-GC)\times BD/ Water density\times{10}^{-4}$ with WC the gravimetric soil water content (%), D the thickness of the soil layer (mm), GC the gravel content (%, in mass), BD the bulk density of the soil layer (g cm^−3^), Water density in gcm-3 and the 10-4 factor to convert ha into m2 |
| L261 superscript -3 | Done |
| L341 use p, not rho, same line 375-379. | We replace all « rho » coefficient with « r » as it indicates Pearson correlation coefficients |
| Table 2: Why not show correlation coefficients for all entries in this table? I get that you want to show the significant correlations, but I don't see why you can't show all of them. Same with figure 2. | To enhance table readability, we prefer to present only statistically significant correlations discussed in the text. |
| Table 4 Asteraceae is spelt wrong. Plantaginae should be Plantaginaceae. | Thanks for the vigilance, the mistakes were corrected (see Table 4) |
| Throughout, use . not , as a decimal point indicator (sorry, American formatting) | Done |
| L409 this is figure 4. | The numerotation problem was solved |
| L416 you say Nitrogen Supply was very close to 0 for all clusters, but then say it was significantly different between clusters 2-1 and 3-1. This feels contradictory? | We reformulated the sentence to be more explicit and avoid the contradiction (l.418-419) |
| L423 This is figure 5. | The numerotation problem was solved |
| L431 I'm not sure you can say this vegetation was spontaneous, given you seeded it? | It’s true : the vegetation was composed of both sown and spontaneous species. We replaced « spontaneous vegetation » by « plant communities » (l.434) |
| L439 should 'assumption' be 'hypothesis'? | We replace « assumption » with « hypothesis » as suggested (l.441) |
| L444 change 'were' to 'have been' to make it sound less like this proof came from the current study. | We did the replacement as suggested (l.446) |
| L464-470 don't seem to add anything to the discussion, but instead just restate what you did in the study. | We improve the discussion of this idea (l.466-480) |
| L472 I'm not convinced that this study does emphasise this need. | We have rephrased this and improved the discussion of this idea (l.466-480) |
| L476 'Bunches' is a curious word to choose. Maybe 'combinations'? | We replaced « bunches » with « combinations » as suggested (l.483) |
| L520 What is 'soluble concentration'? Do you mean 'solute concentration'? | We replaced « soluble concentration » with « water-soluble compound concentration », the correct formulation (e.g. Roumet et al. 2016 about the root econoic spectrum) (l.521,522,524) |
| L521: similarly, what is 'root water-soluble concentration'? | We replaced « soluble concentration » with « water-soluble compound concentration », the correct formulation (e.g. Roumet et al. 2016 about the root econoic spectrum) (l.521,522,524) |
| L555 this is figure 6. (Update numbers within text too on eg L574) I find the acronyms in this make it almost incomprehensible. It feels like there is enough space to fully spell out what each of the acronyms mean. | The numerotation problem was solved. Figure 6 must be synthetic, as it summarises the highlighted relationships. We are convinced that acronyms are better than full names of variables, which are too long. All acronyms are explained in the legend for Figure 6, and most of them are commonly used. |
| L608-611 can be deleted. | Done |
|  |  |
| **Reviewer #4** | |
| The general comment is about the number of references, that I find too high for a research article. Please try to reduce a bit their number, if possible. | It’s true that we cited numerous articles. However, as our research deals with multifunctionality, it necessarily involves references from different domains, which increases the total number of citations. |
| Detailed comments follow here: |  |
| L212: please report if 10 g each are dry matter or fresh matter | Done (l.228) |
| L215: “40C” should be “40°C” | Done (l231) |
| L217-218: “adjacent sieves”. Do you mean the one with the immediately lower and immediately higher mesh size? | We clarified the sentence and referred again to the published methodology (Le Bissonnais 1996) (l.234) |
| L240 and L242: why 103°C and not 105°C that I suppose was the standard oven-drying temperature for gravimetric soil humidity determination? | Thanks for the observation, the soil standard protocol was applied to dry the soil samples (105 °C), it was corrected in the text (l.257, 259) |
| L348-350: please double check because it seems that the trends were wrongly reported. What “only slightly to Water Supply” does it mean? “The relation was positive…”: seems it is the opposite | Thank you for your observations. There are actually several mistakes in this paragraph. We corrected lines 365–368. |
| L359: add also Run-off reduction after Biomass Production | Done (l.378) |
| Table 4: are there any reasons why in the post hoc test column of cluster 2 the statistical letters are reported as capital letters? | No reason. I’s now corrected (Table 4) |
| Table 4 footnote: after “Different letters indicate significant differences” I would add “between clusters. “striking characteristics” should be explain better (e.g., in brackets you could report something like “i.e., characteristics for which the value of the cluster is significantly higher or lower from all the other two clusters”) | Thanks for the suggestion, Table 4 footnote was modified as suggested |
| L391: it is “Figure 4” and not 3 | The numerotation problem was solved |
| L405: it is “Figure 5” and not 4 | The numerotation problem was solved |
| L438: Mg ha-1 of dry matter? | Biomass was measured in dry matter, it was added at the end of the bracket (l.459) |
| L462: replace commas with dots in 0,58 and 0,49 | Done |
| L500: consider to replace “homogeneous plot” with “homogeneous field” | The text was modified as suggested (l.526) |
| L531: it is “Figure 6” and not 5 | The numerotation problem was solved |
| L540-541: please check the presence of too many spaces between words | Done (l.566) |
| L585-586: sounds like a repetition of the first sentence | Thanks for the observation, the mistake was corrected (l.611-613) |
| L595: I would suggest adding also climate to cropping systems and soils | We replaced « soils » by « pedoclimatic conditions » to include both soils and climates (l.621) |
